# Emergence of CTX-M-27-producing *Escherichia coli* of ST131 and clade C1-M27 in an impacted ecosystem with international maritime traffic in South America

**DOI:** 10.1093/jac/dkaa069

**Published:** 2020-03-13

**Authors:** Miriam R Fernandes, Fábio P Sellera, Marcos P V Cunha, Ralf Lopes, Louise Cerdeira, Nilton Lincopan

**Affiliations:** d1 Department of Clinical Analysis, Faculty of Pharmaceutical Sciences, University of São Paulo, São Paulo, Brazil; d2 Department of Internal Medicine, School of Veterinary Medicine and Animal Science, University of São Paulo, São Paulo, Brazil; d3 Department of Pathology, School of Veterinary Medicine and Animal Science, University of São Paulo, São Paulo, Brazil; d4 Department of Microbiology, Institute of Biomedical Sciences, University of São Paulo, São Paulo, Brazil

Sir,

Surveillance studies of ESBL-producing *Escherichia coli* have identified a globally disseminated high-risk clone named ST131, with strains belonging to three clades (A, B and C) and three different subclades (C1, C1-M27 and C2). While C2 is associated with CTX-M-15, clade C1-M27 has been associated with CTX-M-27.^1^ Nowadays, the MDR and CTX-M-27-producing ST131-C1 cluster has been considered a novel epidemic clone.^1–3^ In South America, neither *bla*_CTX-M-27_ nor *E. coli* ST131 C1-M27 have been reported so far.

During a local surveillance study conducted to monitor the presence of WHO critical priority pathogens in impacted marine ecosystems, brown mussels (*Perna perna*) and oysters (*Crassostrea* spp.) were collected from 14 near-shore sites located at different distances from the port of Santos (the largest port of Latin America). Mussel (*n = *10) and oyster (*n = *10) samples, collected from each site, were placed into sterile plastic bags. The samples were kept refrigerated and processed within 3 h after collection. Following standard methods for the examination, 25 g of bivalves were distributed in sterile plastic bags containing 225 mL of Brain Heart Infusion broth and incubated at 37°C for 24 h. Subsequently, the samples were streaked onto MacConkey agar plates supplemented with ceftriaxone (2 mg/L), meropenem (2 mg/L) or colistin (2 mg/L), following incubation at 37°C for 24 h.

Two ceftriaxone-resistant *E. coli* isolates were recovered from mussel (*E. coli* 6M) and oyster (*E. coli* MO) samples collected from two different sites (23.987125S, 46.308609 W and 23.976040S, 46.372580 W) close to the port. Antimicrobial susceptibility testing, performed by disc diffusion and/or Etest methods,^4^^,^^5^ revealed that both strains were resistant to amoxicillin/clavulanic acid, aztreonam, trimethoprim/sulfamethoxazole, ceftiofur (>32 mg/L), ceftazidime (>32 mg/L), cefotaxime (>32 mg/L) and tetracycline. Additionally, *E. coli* MO was resistant to nalidixic acid (>32 mg/L) and ciprofloxacin (>4 mg/L). PCR screening and Sanger sequencing revealed that these isolates were positive for the *bla*_CTX-M-27_ ESBL gene.

*E. coli* strains were subjected to WGS using the Illumina NextSeq (2 × 150 bp) platform (Illumina, USA). *De novo* assemblies were performed using Spades v. 3.11. WGS data were analysed using bioinformatics tools available from the Center for Genomic Epidemiology (www.cge.dtu.dk).

*E. coli* 6M (accession number: NCWA00000000.1) belonged to serotype O86:H18 and sequence type ST38/CC38, whereas *E. coli* MO (accession number: NCVZ00000000.1) belonged to serotype O25b:H4 and ST131/CC131. These STs have been globally disseminated among humans, animals and aquatic environments, being commonly associated with CTX-M variants.^1^^,^^2^^,^^6–10^ Both strains belonged to the high-virulence phylogenetic group B2. In this regard, virulome analysis of *E. coli* 6M revealed the presence of *iss* (increased serum survival), *astA* (EAST-1 toxin), *eatA* (enterotoxigenic autotransporter A), *capU* (hexosyltransferase homologue), *nfaE* (diffuse adherence fibrillar adhesin) and *eilA* (*Salmonella* HilA homologue) genes, whereas *iha* (adherence protein), *sat* (secreted autotransporter toxin), *gad* (glutamate decarboxylase), *senB* (enterotoxin) and *iss* genes were found in *E. coli* MO. Moreover, *E. coli* MO carried *fimH30* (associated with ST131) and the C1 subclade-specific prophage-like region (M27PP1).^11^ In this regard, the virulome content (i.e. *iha*, *sat*, *gad*, *iss* and *senB* genes) of *E. coli* MO was identical to that of other *E. coli* strains of ST131 and C1-M27 clade.^2^^,^^3^ On the other hand, both strains displayed an identical resistome for aminoglycosides (*strA, strB* and *aadA5*), β-lactams (*bla*_CTX-M-27_), sulphonamides (*sul1* and *sul2*), trimethoprim (*dfrA17*) and tetracycline [*tet*(A)], as previously observed in *E. coli* of ST131 and C1-M27;^2,3^ whereas mutations in the quinolone resistance-determining regions of *gyr*A (Ser83Leu, Asp87Asn), *par*C (Ser80Ile, Glu84Val) and *par*E (Ile529Leu) genes were only identified in the *E. coli* MO strain. FIB and FII, and Col156, FIA, FIB and FII replicon types were identified in *E. coli* 6M and MO strains, respectively.

Mobilization of plasmids ∼130 kb in size (named pMO and p6M), bearing *bla*_CTX-M-27_ genes, was achieved by bacterial transformation using *E. coli* TOP10. FIB and FII replicons were identified in p6M (FAB formula F2:A−:B10), whereas FIA, FIB and FII replicon types were confirmed in pMO (FAB formula F1:A2:B20). The complete sequence of the pMO plasmid (GenBank accession no. MG886288) was obtained using *de novo* assembly, followed by gap closure by PCR and Sanger sequencing.

**Figure 1:**
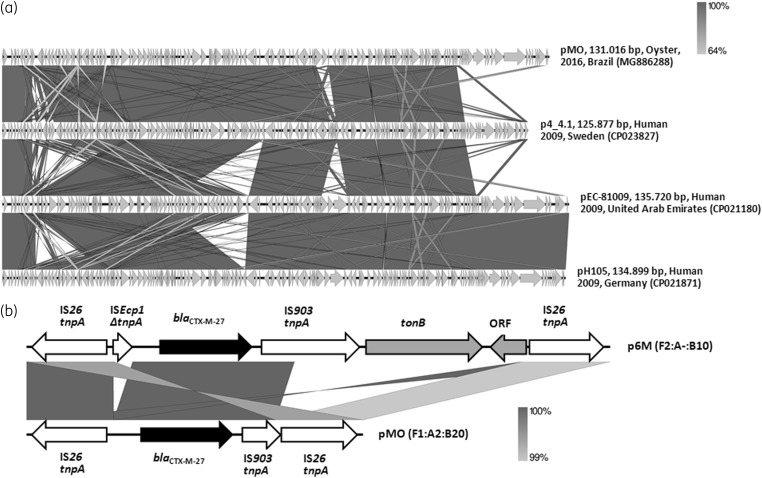
(a) Full-length alignment of four F1:A2:B20 plasmids harboured by CTX-M-27-producing *E. coli* strains of ST131 and clade C1-M27, identified in Brazil (pMO, GenBank accession number: MG886288), Sweden (p4_4.1, 125.877, GenBank accession number: CP023827), the United Arab Emirates (pEC-81009, GenBank accession number: CP021180) and Germany (pH105, 134.899, GenBank accession number: CP021871). Arrows indicate the direction of transcription, whereas the grey bands denote regions of identity. Overall, the identity was more than 95%. (b) Schematic representation of the genetic environment of *bla*_CTX-M-27_ genes carried by the pMO and p6M plasmids identified in CTX-M-27-producing *E. coli* strains, isolated from areas impacted by intensive maritime traffic and transoceanic shipping activities, in Brazil. Arrows indicate the direction of transcription of *bla*_CTX-M-27_ genes (black arrow), genes related to mobile elements (white arrows) and *tonB* (related to energy transduction functions) and ORF genes (grey arrows).

The pMO plasmid was 131 016 bp in length, containing 52.1% GC and 171 coding regions (CDS), of which 129 CDS encoded proteins with known functions (i.e. proteins related to plasmid replication, partition, maintenance, conjugation, toxin–antitoxin systems and antimicrobial resistance). Besides *bla*_CTX-M-27_, the pMO plasmid harboured *aadA5*, *sul1*, *dfrA17*, *tet*(A) and *mphA* resistance genes, similarly to F1:A2:B20 plasmids harboured by the C1-M27 clade (Figure 1a). In fact, pMO showed a high nucleotide identity (>95%) to other F1:A2:B20 plasmids harboured by CTX-M-27-producing *E. coli* strains of ST131 and clade C1-M27, identified in European, Asian and North American countries (Figure 1a), which could support intercontinental dissemination of this sort of plasmid.

Although analysis of the genetic environment of *bla*_CTX-M-27_ genes, carried by both *E. coli* strains, revealed the presence of IS*26* and IS*903* mobile elements, *E. coli* MO presented a truncated IS*903* upstream of the *bla*_CTX-M-27_ gene, whereas *E. coli* 6M presented a truncated IS*Ecp1* downstream of the *bla*_CTX-M-27_ gene, and *tonB* and ORF genes (Figure 1b). 

In summary, to our knowledge, we report the first identification of CTX-M-27-producing *E. coli* strains, of ST131 and clade C1-M27, in Brazil. In this regard, since CTX-M-27-positive *E. coli* strains were recovered from areas impacted by intensive maritime traffic and transoceanic shipping activities, a possible introduction of international clones via commercial shipping routes could be speculated.^12^ Another option could be polluted effluents with previously unnoticed presence of CTX-M-27-positive strains. In fact, in Brazil, aquatic environments receiving large quantities of urban wastewater, animal waste and hospital effluents have been recognized as potential sources for the dissemination of CTX-M- and carbapenemase-producing Enterobacterales.^13^ Therefore, continued monitoring of ESBL-producing *E. coli* in South American countries remains necessary to elucidate the local epidemiology and dynamics of the transmission of high-risk clades with pandemic potential.
